# Late relapse of anti-*N*-methyl-d-aspartate receptor (NMDAR) encephalitis: a case report

**DOI:** 10.1186/s13256-024-04886-5

**Published:** 2024-11-29

**Authors:** Hamidreza Amiri, Mehdi Karimi, Fakhreddin Shariatmadari

**Affiliations:** 1 Student Research Committee, Arak University of Medical Sciences, Arak, Iran; 2 Faculty of Medicine, Bogomolets National Medical University, Kyiv, Ukraine; 3 Department of Pediatrics, Arak University of Medical Sciences, Arak, Iran

**Keywords:** Autoimmune encephalitis, Anti-NMDA encephalitis, Anti-*N*-methyl-d-aspartate receptor encephalitis, Relapsing encephalitis, Anti-NMDA antibody, Case report

## Abstract

**Background:**

Anti-*N*-methyl-d-aspartate receptor encephalitis is a sporadic autoimmune disorder of the brain that presents in a variety of neuropsychiatric manifestations, including seizures, psychosis, and alterations in behavior. *N*-methyl-d-aspartate receptor is primarily seen in young females. Although this disease can be treated, it can relapse in rare cases. Relapsing typically occurs within the early years following the initial episode and is exceedingly rare after 5 years.

**Case presentation:**

In this case study, we report on a 16-year-old Iranian female experiencing a relapse of anti-*N*-methyl-d-aspartate receptor encephalitis 8 years after her initial diagnosis. She was admitted to the hospital with dysphasia (a speech disorder) and dyslexia (reading and writing impairment). A thorough clinical evaluation revealed the presence of anti-glutamate receptor type *N*-methyl-d-aspartate receptor antibodies in her serum and cerebrospinal fluid, confirming the diagnosis. Following treatment with immunotherapy and plasmapheresis, she made a complete recovery.

**Conclusion:**

This case of relapsing anti-*N*-methyl-d-aspartate receptor encephalitis, occurring more than 5 years after the initial episode, is exceptionally rare. This late relapse underscores the importance of long-term follow-up for patients with this condition.

## Introduction

In recent years, anti-*N*-methyl-d-aspartate receptor (NMDAR) encephalitis has garnered increasing recognition as a critical entity in autoimmune neurology. Characterized by its protean manifestations and often challenging diagnostic pathways, this disorder highlights the intricate interplay between immunological dysregulation and central nervous system function. While the acute phase and early relapses of NMDAR encephalitis have been extensively documented, the phenomenon of late relapses remains relatively underexplored. Understanding the clinical features, risk factors, and optimal treatment strategies for late relapse is crucial for healthcare providers managing patients with anti-NMDAR encephalitis. This late relapse highlights the importance of long-term follow-up of these patients.

In this case report study, we presented a case of relapsed anti-NMDAR encephalitis over 8 years after the first episode. This case report underscores the importance of considering anti-NMDA receptor encephalitis in patients with unexplained neurological symptoms, particularly when routine blood tests, medical brain imaging, and electroencephalogram (EEG) results do not reveal abnormalities. In addition, this case report indicates the potential for late recurrence of this disease several years following the first diagnosis. Physicians should consider the late relapse of this disease. This underscores the significance of conducting long-term follow-up with and surveillance of patients with this disease, and in the case of suspicious symptoms, the patient should be checked for recurrence.

## Case presentation

### Case presentation and patient information

A 16-year-old Iranian female was brought to the hospital by her parent with a chief complaint of progressive speech impairment over the past 2 weeks. Initially, she experienced difficulty pronouncing certain letters, which worsened to intermittent stuttering during verbal communication. Over time, she also exhibited impairment in reading and writing. Concurrently, her parents noticed emotional instability, atypical behaviors, and a decline in interest. She occasionally displayed disorientation to time and place, affecting her judgment. Additionally, she developed delusional thoughts, mistaking her imagination for reality, causing significant anxiety.

Although the patient had no relevant family history of neurological or psychiatric disorders, she had a significant past medical history. Her parents stated that, 8 years ago, she experienced similar symptoms to her current presentation, which included a seizure. After a comprehensive examination, she was diagnosed with anti-*N*-methyl-d-aspartate receptor (NMDAR) encephalitis. Then, she was treated with high-dose corticosteroids, immunotherapy, plasmapheresis, and plasma exchange, leading to a complete recovery without lasting neurological issues.

## Clinical findings

### Physical examination

The patient underwent detailed neurological and physical examinations by a specialist. Significant findings included neurological symptoms such as altered mental status, agitation, paranoia, hallucinations, and mood swings. Physical assessments revealed movement disorders such as facial twitching, involuntary writhing, and abnormal muscle contractions, leading to unusual postures. Initially, she exhibited generalized hyperreflexia and increased muscle tone. Cognitive tests showed fluctuating consciousness levels and impaired executive function. Throughout her hospitalization, she developed limb dystonia, indicating advancing neurological impairment.

### Laboratory findings

Comprehensive blood tests were conducted to evaluate the patient’s blood to find any abnormalities and aid in differential diagnosis. This included complete blood counts (CBC), coagulation tests, a serum electrolyte panel, thyroid function tests (TFT), renal function tests (RFT), a comprehensive metabolic panel (CMP), paraneoplastic antibodies, and an autoimmune panel. These tests were requested to evaluate the patient and exclude other potential causes.

CBC, coagulation, and serum electrolyte tests were conducted to evaluate the patient’s blood cells, aiming to rule out infection and inflammation. The results showed no abnormality.

Additionally, RFT and TFT were performed to exclude the possibility of thyroiditis or uremic encephalopathy (UE), which can present with similar symptoms. The results showed no significant abnormalities, indicating these organs’ infection, inflammation, or dysfunction.

Paraneoplastic antibodies and an autoimmune panel (ANCA-c, ANCA-p, anti-phospholipid IgG, anti-NMO, anti-SSA/Ro, anti-SSB/La, anti-dsDNA) were negative, and only serum anti-NMDAR antibodies showed a positive result.

The cerebrospinal fluid (CSF) analysis revealed a slight elevation in white blood cells (pleocytosis) and protein levels but no other significant abnormalities. Notably, the CSF tested positive for anti-NMDAR antibodies, with a titer level of 1000, confirming the presence of these antibodies in the nervous system (Table 1).

**Table 1:** Results of CSF analysis

Test	Normal range	Result	Interpretations
Appearance	Clear/colorless	Clear/colorless	Normal in appearance
Gram stain	Negative	Negative	Normal, no infection
WBC	> 5 cells/µL	8 cells/µL	Mild pleocytosis, indicating inflammation
Glucose	40–57 mg/dL	48 mg/dL	Normal range
Protein	15–45 mg/dL	54 mg/dL	Elevated, suggesting a disrupted blood–brain barrier
LDH	0.3–3.0 U/L	3.9 U/L	Elevated, may indicate inflammation or neuronal damage
Oligoclonal bands	Negative or few (< 5 bands)	Positive (multiple bands)	Reflects intrathecal immune activity
IgG index	0.3–0.7	0.9	Elevated, indicating an intrathecal immune response
IgM index	0.2–0.6	0.3	Normal range
NMDA receptor antibodies	Negative	Positive (titer: 1000)	Confirmatory of anti-NMDA receptor encephalitis

*WBC* white blood cells, *LDH* lactate dehydrogenase, *Ig* immunoglobulin

### Diagnostic medical imaging

To examine the patient more closely, computed tomography (CT) scan and magnetic resonance imaging (MRI) of the brain; chest X-ray (CXR); ultrasound of the thyroid, abdomen, and pelvis; and electroencephalogram (EEG) tests were requested and performed.

In thyroid ultrasound, both thyroid lobes were normal with homogeneous parenchymal echo and without solid-cystic lesions, and no abnormal findings were seen. In the uterus and ovary ultrasound, the uterus and ovaries had normal echo and dimensions, and there was no evidence of cystic or solid lesions.

**Fig. 1:**
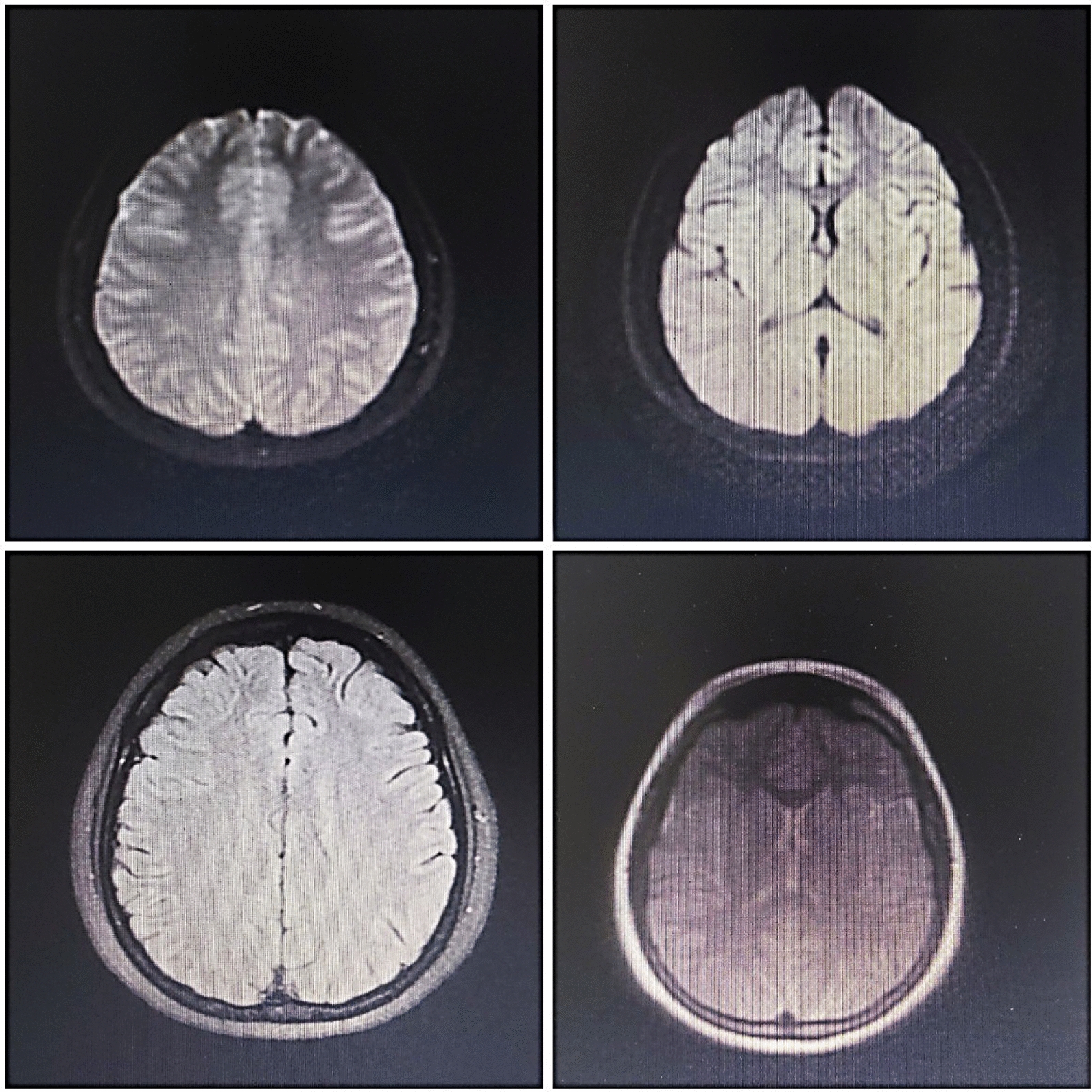
Magnetic resonance imaging findings. Brain magnetic resonance imaging sequences revealed no suspicious signal alterations a the time of diagnosis

**Fig. 2:**
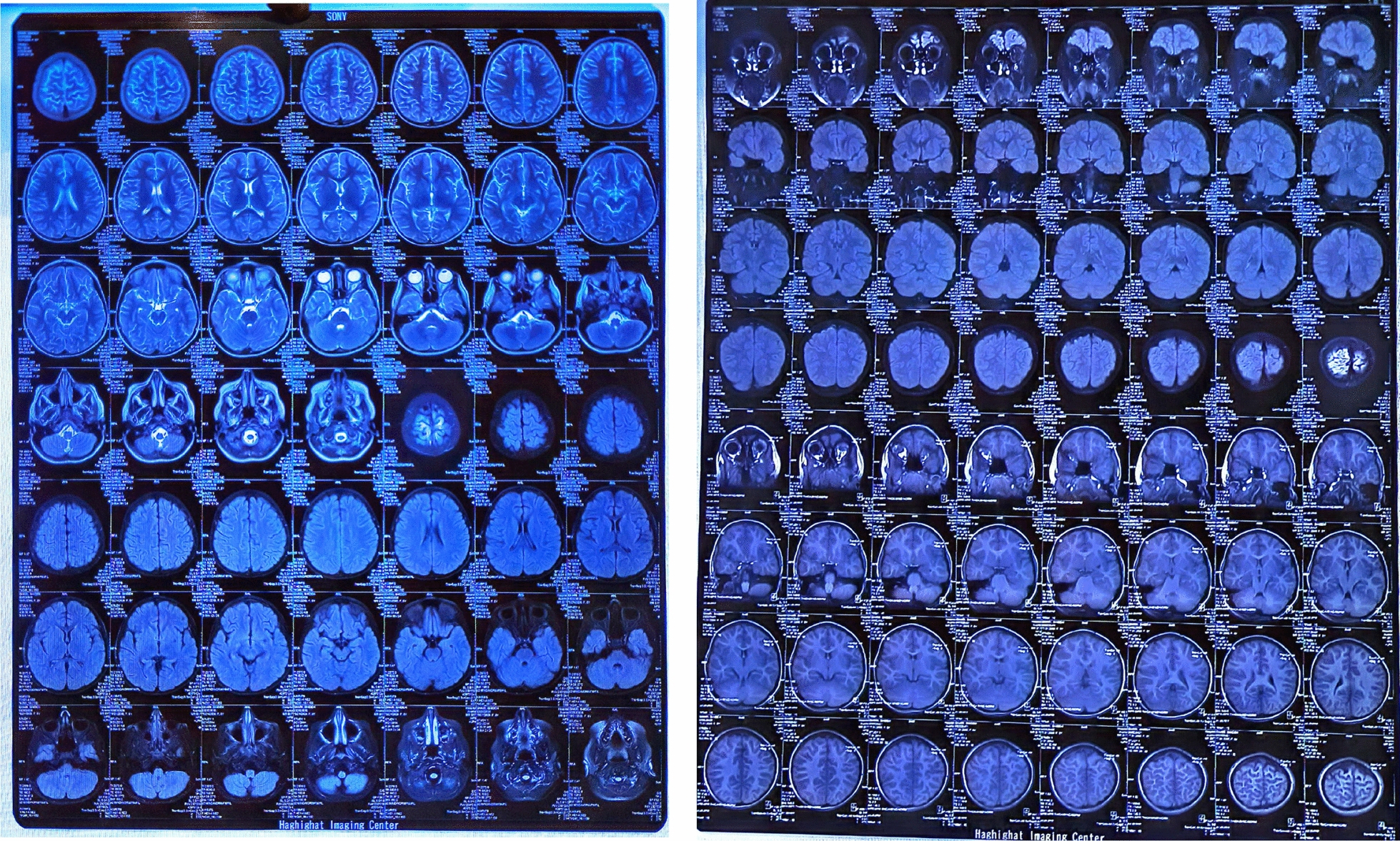
Brain magnetic resonance imaging (MRI)

**Fig. 3:**
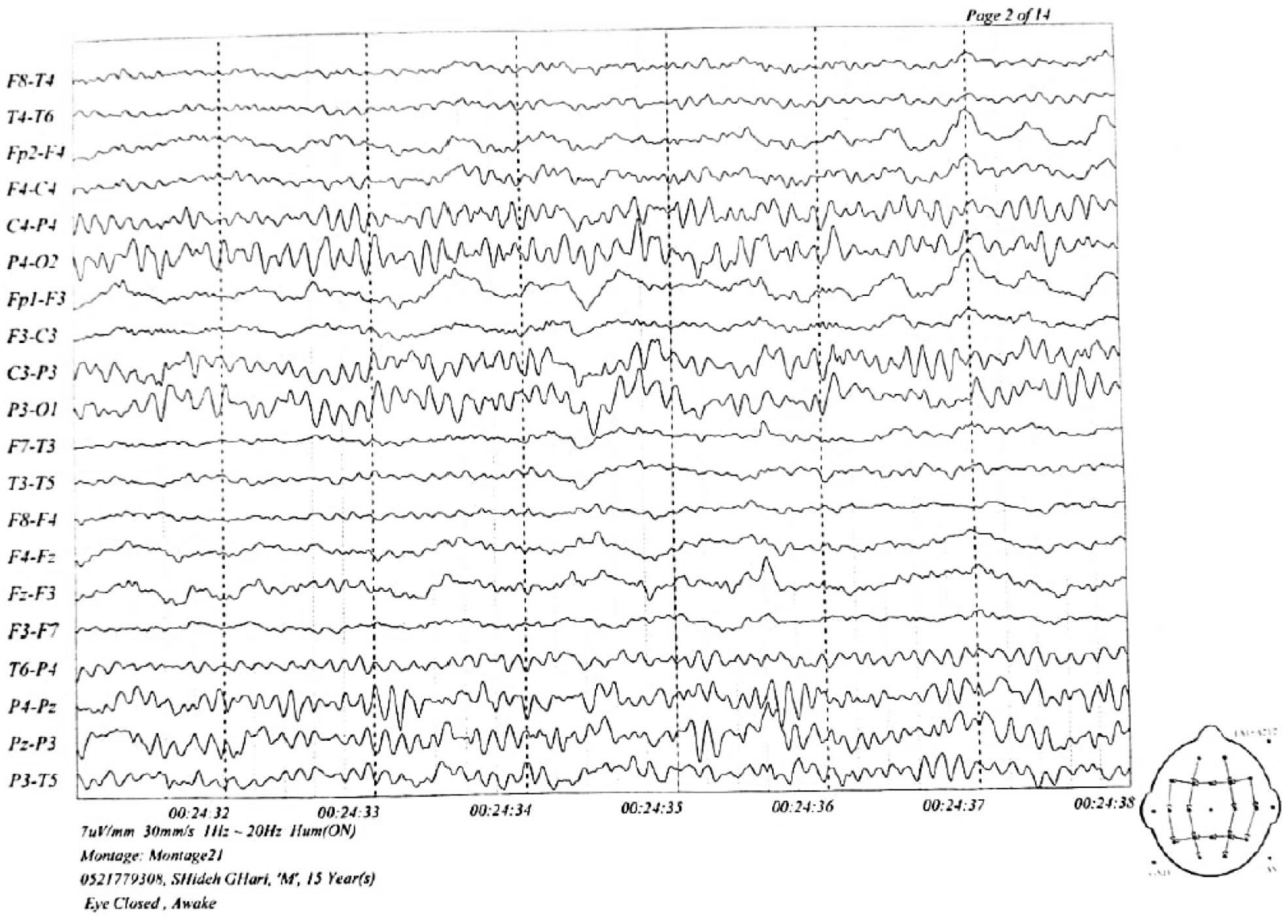
Electroencephalogram (EEG) findings

The MRI findings were normal, but several diffuse cortical hyperintensities were observed, consistent with the patient’s history of anti-NMDAR encephalitis. This may indicate an inflammatory process related to a relapse. However, the overall normal findings and the absence of abnormalities in complementary imaging studies, including brain CT, chest X-ray, and ultrasounds, suggest no concurrent structural or systemic pathology. The EEG was also evaluated and found normal (Figs. 1, 2, 3).

## Diagnosis

Finally, on the basis of the clinical findings (signs and symptoms, medical history of patients), medical imaging results, and laboratory findings (antibodies detected in the serum and CSF, blood analysis), this patient was diagnosed with “late relapse of anti-*N*-methyl-d-aspartate receptor (NMDAR) encephalitis.”

We encountered several diagnostic challenges. The recurrence of symptoms years after the initial episode complicated timely recognition, especially when routine tests and imaging showed no abnormalities. This situation required a high level of clinical suspicion and comprehensive testing. Additionally, identifying the NMDAR autoantibody presented its own difficulties, as our facility lacked the necessary resources, necessitating the time-consuming process of sending samples to another center.

## Treatment

The patient underwent treatment with a combination of immunotherapy, symptomatic management, and supportive care. Immunotherapy reduces inflammation and modulates the immune system.

As the first line of treatment, she received intravenous methylprednisolone (IVMP) and intravenous immunoglobulin (IVIG) for five consecutive days. After being administered these drugs for 5 days, the patient was only slightly better, but there was no satisfactory improvement in her symptoms.

### Plasmapheresis

The patient underwent 10 plasmapheresis sessions—initially, 5 sessions were administered daily, followed by 5 sessions spaced every other day—to help remove the harmful NMDA antibodies from her serum. Her vital signs and overall condition were carefully monitored throughout each session to ensure her safety. After the sixth session, her symptoms showed some improvement. We also conducted blood tests before and after each session to track the treatment’s effectiveness and monitor her electrolyte levels. Following each session, the patient was observed for any immediate adverse effects, such as low blood pressure, bleeding, or allergic reactions. Her neurological status was regularly assessed, and laboratory tests, including antibody titers and imaging, were used to evaluate her prognosis.

As part of her treatment, she also received a low-dose prednisolone at 20 mg per day, which was gradually reduced by 5 mg each week over the course of a month and then discontinued. The patient tolerated the treatment well, with no adverse effects observed.

Table 2 demonstrates the treatment regimen of the patients.

**Table 2:** Treatment regimen of anti-NMDAR encephalitis

Medications	Dosage	Duration/total dose
IVMP	1 g/day	5 days (5 g in 5 days)
IVIG	24 g/day	5 days (120 g in 5 days)
Plasmapheresis	Exchange 1.5 plasma volumes per session	10 sessions
Prednisolone	20, 15, 10, and 5 mg/day	4 weeks (gradual reduction by 5 mg every week)

IVMP, intravenous methylprednisolone; IVIG, intravenous immunoglobulin

## Follow-up of the patient

**Fig. 4:**
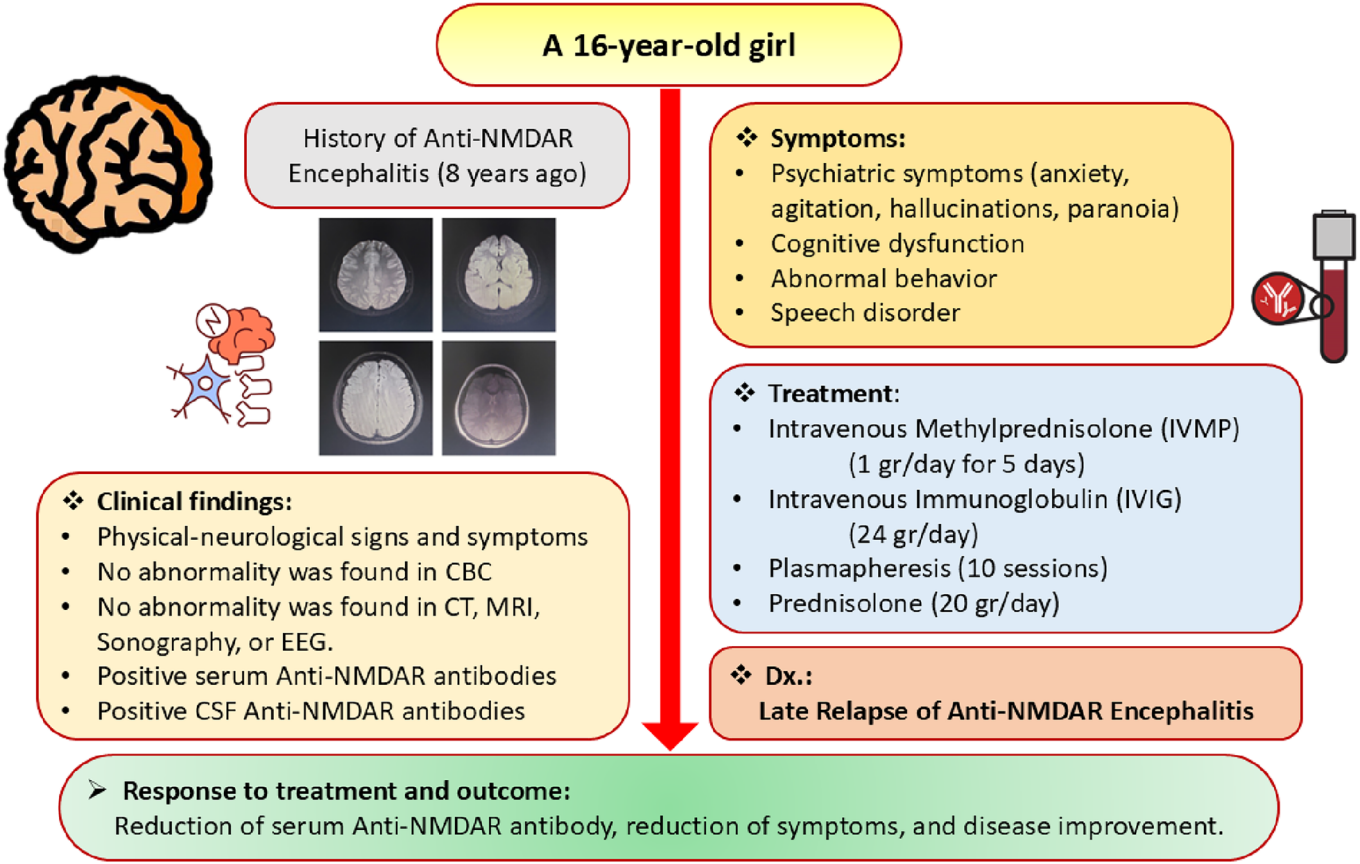
A summary of the case

After the patient was discharged from the hospital, her neurologists and immunologists regularly followed up with her. She underwent periodic neurocognitive assessments to evaluate her recovery and her need for continued rehabilitation. Additionally, her CBC, LFT, and RFT were periodically monitored. Finally, her serum anti-glutamate receptor NMDA decreased, and the symptoms and signs improved ultimately approximately 40 days after starting the treatment. Figure 4 demonstrates a summary of this female patient.

## Review of anti-NMDAR encephalitis

Anti-*N*-methyl-d-aspartate receptor (NMDAR) encephalitis is a rare autoimmune disease characterized by immune-mediated depletion of NMDA glutamate receptors. It leads to a spectrum of progressive neuropsychiatric symptoms ranging from psychosis to seizures and cognitive impairment [1]. It is the most common form of autoimmune encephalitis in which antibodies are produced against the NMDA receptor [2–4] and may affect 1 in 1.5 million people annually [5].

This disease has significant neurological and psychiatric implications [6]. It can cause a clinical syndrome with memory and cognitive disturbances, speech disorders, psychosis, abnormal behavior, irritability, movement disorders, seizures, impaired consciousness, and even weight loss. While the clinical course typically involves an acute phase followed by gradual improvement with immunotherapy, late relapses can occur, posing a particular challenge for diagnosis and treatment [2, 7]. It has been observed that approximately one in six patients may encounter a relapse of the disease [8]. Most patients experience an initial relapse during the first 2 years [9], and relapses are rare 5 years after the first episode [10].

### Pathophysiology

Anti-*N*-methyl-d-aspartate receptor (NMDAR) encephalitis is characterized by autoimmune processes targeting the NR1 subunit of NMDA receptors, leading to profound neurologic dysfunction. The primary pathophysiological mechanism involves autoantibody-mediated internalization of NMDA receptors triggered by binding to the NR1 subunit [4]. This reduces the number of functional receptors crucial for glutamatergic signaling and synaptic plasticity. These autoantibodies also disrupt the interaction between NMDA receptors and ephrin-B2 receptors (EPHB2R), exacerbating receptor internalization. The disease pathogenesis further entails complex immune responses: B cells produce pathogenic antibodies facilitated by elevated levels of B cell activating factor (BAFF), while CD4+ and CD8+ T cells support this process [11]. Dysregulation of cytokines and chemokines, such as elevated pro-inflammatory cytokines (IL-6 and TNF-α) and chemokines (CXCL10 and CXCL13), along with disrupted anti-inflammatory responses (IL-10 and IL-13), contribute to blood–brain barrier disruption and immune cell infiltration into the CNS. This inflammatory milieu and neuronal receptor loss lead to altered synaptic function, impaired plasticity, and diverse neuropsychiatric symptoms. Understanding these intricate mechanisms is crucial for developing targeted therapies aimed at improving outcomes for patients with anti-NMDAR encephalitis. [1, 4, 11, 12].

### Diagnosis

The diagnostic criteria for anti-NMDAR encephalitis divide cases into two groups, “probable” and “definite,” depending on the various clinical and laboratory parameters [13]. The disease can be easily diagnosed by detecting antibodies in the blood or cerebrospinal fluid (CSF). Neuroimaging may show no abnormalities, and antibody test results may take several weeks to confirm [14]. The clinical diagnostic criteria are reliable in determining the initiation of immunomodulatory therapy in cases that positively meet the criteria [15].

### Treatment

The initial treatment for anti-NMDAR encephalitis involves primary immunotherapy, typically combining high-dose steroids, immunoglobulins, and plasma exchange. If this proves ineffective, secondary immunotherapy with rituximab and immunosuppressants is recommended to enhance results and prevent recurrence. For relapses, first-line treatments include intravenous methylprednisolone at 1 g per day for 3–5 days and intravenous immunoglobulin at 2 g/kg of body weight. If the response is inadequate, second-line treatments with rituximab, a monoclonal antibody targeting CD20 on B cells, and cyclophosphamide, an immunosuppressant, are used in severe or refractory cases [1, 16–18].

### Prognosis

While anti-NMDAR encephalitis is a severe condition, timely identification and treatment typically result in favorable outcomes for most patients. Effective early management generally leads to positive long-term effects, although some individuals may endure persistent symptoms or relapses requiring continual treatment. Prognosis varies: approximately 75% of patients recover entirely or experience minor deficits, whereas 25% may suffer severe disabilities. The mortality rate for anti-NMDAR encephalitis is between 8% and 10%. The primary triggers are believed to be tumors or viruses that disrupt the blood–brain barrier via immune and inflammatory responses. Early detection and treatment play a crucial role in improving patient outcomes [1, 19, 20].

## Discussion

Prior research has indicated that a considerable fraction of individuals diagnosed with anti-NMDA receptor encephalitis may relapse, and early detection and treatment of the disease are crucial to prevent relapses [16]. The relapse rate has been reported to be between 8% and 36.4% [8, 9]. These relapses mainly occur in the first 1 or 2 years. Several factors can affect the recurrence of the disease. Female patients may have an increased susceptibility to recurrence [9], as well as delays in receiving therapy [9, 21, 22]. Immunotherapy at the first episode reduces the risk of relapses [23].

**Fig. 5:**
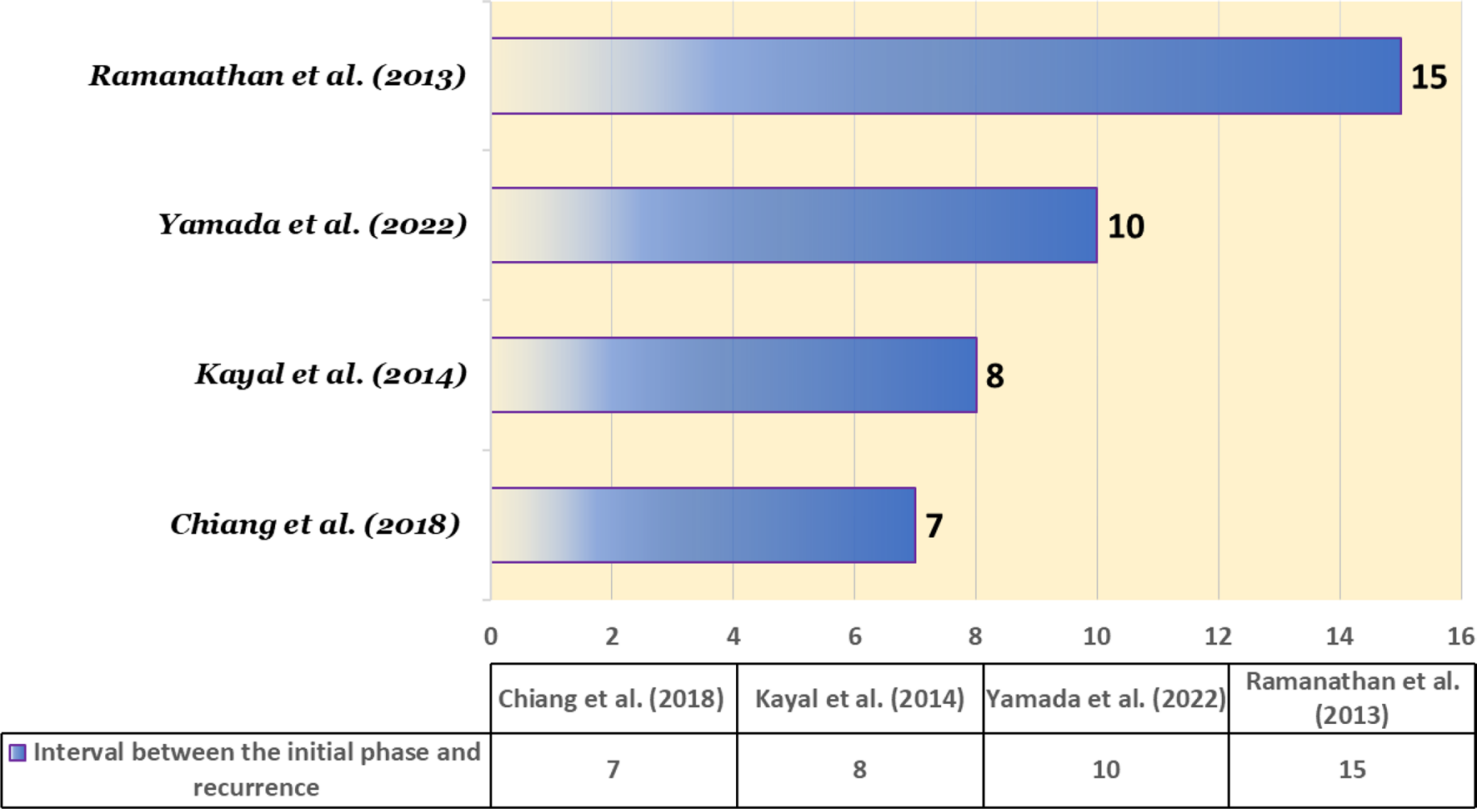
The interval between the initial phase and recurrence in previous case reports

A study by Gong *et al*. showed that 82% of relapses occurred within the initial 2-year period [9]. Few cases have been reported to have a more than 5-year gap between the first and the last episode [23, 24] (Fig. 5). However, in most cases, the severity of the disease in the relapse is less than in the initial phase [8, 9, 25]. In some cases, the relapse can be very severe [26].

Table 3 presents a comprehensive analysis of some late relapses of anti-NMDAR encephalitis.

**Table 3:** Late relapse of anti-NMDA receptor encephalitis in literature

Study	Country	Sex	Age (years)	Late relapse	Prodrome	Symptoms in the recurrence	Treatment and outcome
Kayal *et al*. (2014) [27]	India	Female	13	8 years	Subacute onset anti-epileptic drugs-resistant seizures; unusual behavior; and abnormal body movements	Dysgraphia, tremor of the right hand, seizures, agitation, and anxiety, with lack of response and verbal mutism, bruxism, dystonia, generalized rigidity, insomnia, and depression	IVMP, IVIG, and oral prednisolone Improvement and relief of symptoms The patient had amnesia, minor linguistic disintegration, and intermittent agitation
Yamada *et al*. (2022) [28]	Japan	Female	13	10 years	Lack of sensation in the left hand, decreased level of consciousness, and seizures	Hypoesthesia on the right-side of the body, dysarthria, amusia, and seizures	IVMP and high-dose IVIG Improvement and relief of symptoms Positive outcomes
Chiang *et al*. (2018) [10]	US	Female	33	7 years	Refractory epilepsy	Vomiting and abnormal behavior (due to recent cannabis consumption)	IVMP and rituximab Non-focal follow-up evaluation Positive outcomes
Ramanathan *et al*. (2013) [29]	Australia	Female	31	15 years	Viral prodrome, generalized seizures, psychosis, and excited catatonia	Same as the initial phase	IVMP, immunoglobulin, plasma exchange, and rituximab No long-term improvement Positive outcomes

Prodrome (early sign and symptom); late relapse (interval between the initial phase and recurrence of disease). IVMP, intravenous methylprednisolone; IVIG, intravenous immunoglobulin

In summary, this case report highlights the potential occurrence of delayed relapse in individuals diagnosed with anti-NMDA receptor encephalitis, thereby emphasizing the importance of extended monitoring and long-term follow-up. The management of a late relapse of anti-NMDAR encephalitis is multifaceted, involving re-confirmation of diagnosis, aggressive immunotherapy, symptomatic management, and supportive care. Long-term immunosuppression and regular follow-up are critical to prevent further relapses and manage persistent or new symptoms. Collaboration among neurologists, immunologists, psychiatrists, and other healthcare professionals is essential for optimal patient outcomes. Considering that reported cases of late relapse of anti-NMDAR encephalitis worldwide are scarce, our suggestion for future studies is to explore the occurrence of this disease and its underlying factors in diverse geographical regions.

## Key clinical message

Anti-NMDAR encephalitis, characterized by varied neuropsychiatric symptoms, can relapse years after initial recovery, as highlighted by a case of an 8-year delayed recurrence. This underscores the need for long-term follow-up and vigilance in patients with a history of this condition to ensure timely diagnosis and treatment of relapses.

## Conclusion

Recurrent anti-NMDA receptor encephalitis, though rare, should be considered in patients presenting with neuropsychiatric symptoms, especially those with a previous history of the condition. Early recognition and treatment are crucial for a favorable outcome. Further research is necessary to understand the long-term prognosis and optimal management strategies for recurrent cases.

## Data Availability

Not applicable.

## References

[1] Wang H, Xiao Z. Current progress on assessing the prognosis for anti-*N*-methyl-d-aspartate receptor (NMDAR) encephalitis. Biomed Res Int. 2020;2020:7506590. 32352007 10.1155/2020/7506590 PMC7178504

[2] Dalmau J, Armangué T, Planagumà J, Radosevic M, Mannara F, Leypoldt F, *et al*. An update on anti-NMDA receptor encephalitis for neurologists and psychiatrists: mechanisms and models. Lancet Neurol. 2019;18(11):1045–57. 31326280 10.1016/S1474-4422(19)30244-3

[3] Samanta D, Lui F. Anti-NMDAR encephalitis. StatPearls. Treasure Island (FL): StatPearls Publishing Copyright^©^ 2024, StatPearls Publishing LLC; 2024. 31869136

[4] Ding H, Jian Z, Stary CM, Yi W, Xiong X. Molecular pathogenesis of anti-NMDAR encephalitis. Biomed Res Int. 2015;2015: 643409. 26221602 10.1155/2015/643409 PMC4499418

[5] Hiesgen J, Schutte C. Autoimmune encephalitis: part 1 (epidemiology, pathophysiology and clinical spectrum). S Afr Med J. 2023;113(3):116–21. 36876355 10.7196/SAMJ.2023.v113i3.780

[6] Dalmau J, Graus F. Antibody-mediated encephalitis. N Engl J Med. 2018;378(9):840–51. 29490181 10.1056/NEJMra1708712

[7] Han Y, Gong S, Wan Y, Fu X, He E, Liu M, *et al*. Case report: anti-NMDA receptor encephalitis manifesting as rapid weight loss and abnormal movement disorders with alternating unilateral ptosis and contralateral limb tremor. Front Immunol. 2022;13: 971514. 36189268 10.3389/fimmu.2022.971514 PMC9520482

[8] Feng J, Yang M, Cui D, Huang Z, Ji T, Lian Y. Recurrence of anti-*N*-methyl-d-aspartate receptor encephalitis: a cohort study in central China. Front Neurol. 2022;13: 832634. 35356456 10.3389/fneur.2022.832634 PMC8959942

[9] Gong X, Chen C, Liu X, Lin J, Li A, Guo K, *et al*. Long-term functional outcomes and relapse of anti-NMDA receptor encephalitis: a cohort study in Western China. Neurol-Neuroimmunol Neuroinflamm. 2021;8(2). 10.1212/NXI.0000000000000958 PMC8105891 33589542

[10] Chiang S, Garg T, Hu A, Amin H, Davalos-Balderas A, Alfradique-Dunham I, *et al*. Pearls & Oy-sters: relapse of anti-NMDA receptor encephalitis after prior first-and second-line immunotherapy. Neurology. 2018;90(20):936–9. 29759996 10.1212/WNL.0000000000005517

[11] Ma Y, Wang J, Guo S, Meng Z, Ren Y, Xie Y, *et al*. Cytokine/chemokine levels in the CSF and serum of anti-NMDAR encephalitis: a systematic review and meta-analysis. Front Immunol. 2022;13:1064007. 36761173 10.3389/fimmu.2022.1064007 PMC9903132

[12] He S, Sun C, Zhu Q, Li L, Huang J, Wu G, *et al*. A juvenile mouse model of anti-*N*-methyl-d-aspartate receptor encephalitis by active immunization. Front Mol Neurosci. 2023;16:1211119. 37790883 10.3389/fnmol.2023.1211119 PMC10544982

[13] Potorac A, Varlas VN, Borș RG, Baroș A, Cirstoiu M. The management and diagnosis of anti-NMDA receptor autoimmune encephalitis in pregnant women: a case report and literature review. Medicina. 2023;59(12):2110. 38138213 10.3390/medicina59122110 PMC10744478

[14] Barry H, Byrne S, Barrett E, Murphy KC, Cotter DR. Anti-*N*-methyl-d-aspartate receptor encephalitis: review of clinical presentation, diagnosis and treatment. BJPsych Bull. 2015;39(1):19–23. 26191419 10.1192/pb.bp.113.045518 PMC4495821

[15] Nishida H, Kohyama K, Kumada S, Takanashi JI, Okumura A, Horino A, *et al*. Evaluation of the diagnostic criteria for anti-NMDA receptor encephalitis in Japanese children. Neurology. 2021;96(16):e2070–7. 33653900 10.1212/WNL.0000000000011789

[16] Nguyen L, Wang C. Anti-NMDA receptor autoimmune encephalitis: diagnosis and management strategies. Int J Gen Med. 2023;16:7–21. 36628299 10.2147/IJGM.S397429 PMC9826635

[17] Shin YW, Lee ST, Park KI, Jung KH, Jung KY, Lee SK, *et al*. Treatment strategies for autoimmune encephalitis. Ther Adv Neurol Disord. 2018;11:1756285617722347. 29399043 10.1177/1756285617722347 PMC5784571

[18] Halliday A, Duncan A, Cheung M, Boston RC, Apiwattanakul M, Camacho X, *et al*. Second-line immunotherapy and functional outcomes in autoimmune encephalitis: a systematic review and individual patient data meta-analysis. Epilepsia. 2022;63(9):2214–24. 35700069 10.1111/epi.17327 PMC9796249

[19] Koparal B, Çiçek S, Taner ME, Kuruoğlu A. A rapidly progressive case of anti-NMDAR encephalitis with primary psychiatric symptoms. Psychiatr Danub. 2021;33(2):177–9. 34185739 10.24869/psyd.2021.177

[20] Yu Y, Wu Y, Cao X, Li J, Liao X, Wei J, *et al*. The clinical features and prognosis of anti-NMDAR encephalitis depends on blood brain barrier integrity. Mult Scler Relat Disord. 2021;47: 102604. 33130468 10.1016/j.msard.2020.102604

[21] Titulaer MJ, McCracken L, Gabilondo I, Armangué T, Glaser C, Iizuka T, *et al*. Treatment and prognostic factors for long-term outcome in patients with anti-NMDA receptor encephalitis: an observational cohort study. Lancet Neurol. 2013;12(2):157–65. 23290630 10.1016/S1474-4422(12)70310-1 PMC3563251

[22] Zhong R, Chen Q, Zhang X, Zhang H, Lin W. Relapses of anti-NMDAR, anti-GABABR and anti-LGI1 encephalitis: a retrospective cohort study. Front Immunol. 2022;13: 918396. 35757705 10.3389/fimmu.2022.918396 PMC9218051

[23] Gabilondo I, Saiz A, Galán L, González V, Jadraque R, Sabater L, *et al*. Analysis of relapses in anti-NMDAR encephalitis. Neurology. 2011;77(10):996–9. 21865579 10.1212/WNL.0b013e31822cfc6b

[24] Gresa-Arribas N, Titulaer MJ, Torrents A, Aguilar E, McCracken L, Leypoldt F, *et al*. Antibody titres at diagnosis and during follow-up of anti-NMDA receptor encephalitis: a retrospective study. Lancet Neurol. 2014;13(2):167–77. 24360484 10.1016/S1474-4422(13)70282-5 PMC4006368

[25] Zeng W, Cao L, Zheng J, Yu L. Clinical characteristics and long-term prognosis of relapsing anti-*N*-methyl-d-aspartate receptor encephalitis: a retrospective, multicenter, self-controlled study. Neurol Sci. 2021;42:199–207. 32601745 10.1007/s10072-020-04482-7 PMC7820183

[26] Nakajima H, Unoda K, Hara M. Severe relapse of anti-NMDA receptor encephalitis 5 years after initial symptom onset. Eneurologicalsci. 2019;16: 100199. 31384674 10.1016/j.ensci.2019.100199 PMC6661405

[27] Kayal AK, Das M, Bhowmick S, Synmon B. Relapsing anti-NMDAR encephalitis after a gap of eight years in a girl from North-East India. Ann Indian Acad Neurol. 2014;17(3):349–51. 25221411 10.4103/0972-2327.138526 PMC4162028

[28] Yamada N, Kuki I, Hattori T, Yamamoto N, Nagase S, Nukui M, *et al*. Late relapse of anti-*N*-methyl-d-aspartate receptor encephalitis with amusia and transiently reduced uptake in (123)I-iomazenil single-photon emission computed tomography. Brain Dev. 2022;44(8):558–61. 35662527 10.1016/j.braindev.2022.05.003

[29] Ramanathan S, Mohammad SS, Brilot F, Dale RC. Autoimmune encephalitis: recent updates and emerging challenges. J Clin Neurosci. 2014;21(5):722–30. 24246947 10.1016/j.jocn.2013.07.017

